# Frequency of gallstones and mean BMI in decompensated cirrhosis

**DOI:** 10.11604/pamj.2018.30.123.12742

**Published:** 2018-06-13

**Authors:** Ayesha Aslam Rai, Aisha Nazeer, Nasir Hassan Luck

**Affiliations:** 1 Department of Hepatogastroenterology, Sindh Institute of Urology and Transplantation, Karachi, Pakistani

**Keywords:** End stage liver disease, cholelithiasis, body mass index

## Abstract

**Introduction:**

The aim of the study was to determine the frequency of gallstones in patients with decompensated cirrhosis and to know about mean Body mass index (BMI) in patients of decompensated cirrhosis i.e End stage liver disease (ESLD) with and without gallstones.

**Methods:**

it is a cross sectional descriptive study, conducted at the department of Hepato-gastroenterology, Sindh Institute of Urology and Transplantation (SIUT), Karachi from 1^st^ August 2014 to 28 February 2015. Two hundred patients were enrolled from outpatient clinics of Hepato-gastroenterology that fulfilled the defined selection criteria. Questionnaire was filled for data collection. SPSS version 20.0 was used to analyze data. Mean value of age and BMI was calculated by mean ± S.D. values. Mean ± SD was also calculated for BMI in patients with and without gallstones. Stratification of the age, gender, and liver disease severity were done and chi-Square test was applied. p-values less than 0.05 considered statistically significant.

**Results:**

Two hundred consecutive patients were enrolled among them 112(56%) were male. Mean age was 46.89 ± 11.9, BMI 23.59 ± 4.7 and CTP score was 9.7 ± 1.9. Most of the patient had Child class 'B' cirrhosis 102(51%), most common etiology was found to be Hepatitis C 133 (66.5%), cholelithiasis was found in 59(29.5%), sludge in 36 (18%) and both stone and sludge in 24(12%) of the cases. Advanced liver disease that is, more CTP score and child class 'C' was associated with increased frequency of gall stone formation (p-value = 0.012), and advancing age on age stratification (p-value = 0.024) however no relation was observed with increase BMI, gender, ethnicity, cause or duration of disease in this population.

**Conclusion:**

Gallstone formation is associated with advanced stage of cirrhosis and hepatitis C Virus related CLD, contrary to the established risk factors, no relation of gender or BMI was found in decompensated liver disease.

## Introduction

Cirrhosis is considered a major health problem causing significant morbidity and mortality worldwide [1-3]. In Pakistan cirrhosis is mostly caused by chronic Hepatitis C (28%) and Hepatitis B (22%) infections but alcoholic liver disease is common globally [4]. Gall stones are among the most common cause of gall bladder pathology. It could be cholesterol or pigment stones or mixed. Cholesterol stones occur mostly solitary and are more common in western countries, whereas pigment stones are commonly found in Asian people and are usually, multiple in number [5]. About one third of cirrhotic patients have gallstones, and most of them have pigmented stones [6]. The prevalence of gall stones in cirrhosis is reported to be around 23% with annual incidence of 3.4% [7], whereas in general population it was found to be 10-30% [8-10]. Gall stone formation is associated with many risk factors. Its prevalence increases linearly with advancing age, female gender, pregnancy, multiparity, undiagnosed diabetes, glucose intolerance, viral etiology and prolonged duration of cirrhosis, H. pylori infection, heavy ethanol intake, carbohydrate rich diet, gall bladder wall thickness (>4mm) and portal hypertension [8-15]. Higher Body Mass Index (BMI) is also an independent risk factor for gall stone formation [16]. The increased frequency of gall stones in fertile women is primarily attributed to estrogens that cause excessive secretion of cholesterol into bile, whereas gall bladder stasis and autonomic neuropathy are mainly implicated in advanced cirrhosis. A study done at Taiwan by Li et al concluded that there's no difference in the mean BMI of patients with gallstones (22.2±0.6) and without gallstones (23.7 ± 0.5) [17] in liver cirrhosis and it included patients of both compensated and decompensated cirrhosis, he also concluded that risk of gallstone formation increases with severity of cirrhosis, that is most of the patient having gallstones belongs to child class 'C', in this study sample size was also not calculated, so the results cannot be generalized to patients with decompensated cirrhosis. No local study have been done so there is a need of further study in this respect. The rationale of this study is to assess the frequency of gallstones and Mean BMI specifically in patients with or without gallstones decompensated cirrhosis, in order to ascertain the magnitude of disease, possible relation to BMI.

## Methods

This descriptive cross sectional study was conducted in the out patient department of Department of Hepatogastroenterology, SIUT, Karachi from 1^st^ August 2014 to 28^th^ February 2015. Patient diagnosed as case of decompensated cirrhosis having child Turcotte Pugh (CTP) score of 7 or more were enrolled. BMI was calculated by using formula BMI = Weight in Kg/Height in m^2^. Sample size was calculated by using WHO calculator, taking prevalence = 24.3% [1], precision (d) = 7% and Confidence interval (CI), sample size was 200. Patient aged 30-70 years, had decompensated cirrhosis for more than 6 months were included. While those patients who had active variceal bleed, past history of cholecystectomy, choledochotomy, hemolytic anemia, abdominal or other malignancies, history of tuberculosis, Diabetes Milletus, renal failure, psychiatric illness, pregnancy, using estrogen pills, fibrates, cephalosporins or total paraenteral nutrition were excluded. All the patients were subjected to clinical, biochemical criteria and radiological evaluation. Venous blood samples were collected for prothrombin time, serum albumin and bilirubin. Transcutaneous abdominal ultrasound was performed using 60 Hz device (Model TOSHIBA SSA-520A). Performa was used for data collection, above mentioned ultrasound machine and single laboratory was employed in order to prevent laboratory bias. Outcome variables were mean BMI and presence or absence of gallstones in decompensated cirrhosis. Data was subjected to statistical analysis on SPSS version 20.0. Mean value of age and BMI was calculated by mean ± S.D. values. Mean ± SD was also be calculated for BMI in patients with and without gallstones. Presence of gall stones, number of gall stones (single, multiple) gender and Child Turcot Pugh class, ethnicity was be presented by their frequencies and percentages. Outcome variables are mean BMI and presence or absence of gallstones in decompensated cirrhosis, as mentioned in operational definition. Effect modifiers were controlled through stratification of number of gall stones, gender, ethnicity, age, duration and classes of decompensated cirrhosis (B & C) to see the impact of these on outcome variables. Post stratification Chi- Square test was applied, p- value ≤ 0.05 was taken as significant.

## Results

The total number of patients enrolled were 200, out of which 112 (56%) were male. Table 1 showed basic demographic data of patients, mean age of patients with end stage liver disease (ESLD) was found to be 46.8 ± 11.9 years, most of these were in CTP class B 102 (56%) and mean CTP score was found to be 9.7 ± 1.9 while median CTP score was found to be 9. Overall calculated mean BMI was 23.59 ± 4.7. Most of the patients included in analysis belonged to ethnic group who spoke Urdu language 75(37.5%), followed by Sindhi 55(27.5%) and Punjabi speaking 31(15.5%). Majority of the patients with BMI > 23.59 belonged to Pathan ethnicity, 11 out of 19(57.9%), followed by Balochi 5 out of 9 (55.6%). However, gall stones were more frequently found among Urdu speaking, 25 out of 75(33.3%), followed by Sindhi speaking 16 out of 55(29%). Overall viral etiology was found to be commonest cause of CLD out of which Hepatitis C was found to be the commonest causative agent in study population that is 133(66.5%), followed by cryptogenic CLD and autoimmune related CLD in 21(10.5%) and 9(4.5%) respectively as shown in Table 1. Out of total observed patients 141(70.5%) had no stones while multiple stones were seen in 40(20%) and single in 19(9.5%). Sludge was present in 36(18%) patients, both sludge and stones were observed in 24(12%) patients. Table 2demonstrated stratification of data with respect to gallstones and 46 (34.5%) patients with anti HCV positive were found to have gallstones as compared to only 13(19.5%) of anti HCV negative had gall stones with significant p-value of 0.032. Significant association was also found for CTP score and CTP class, i.e. 0.013 and 0.013 respectively while no association of gallstones could be elicited for age, gender, BMI, duration of disease and ethnicity. Statistically significant association was found for CTP score and CTP class with respect to number of gallstones also, that is p-value of 0.022 and 0.022 respectively, as demonstrated in Table 3. Data was also stratified with respect to mean BMI along with all effect modifiers and it was found to be significant only with Anti HCV (p-value = 0.03), while no association could be elicited for other parameters age, gender, number of gallstones, duration of disease, CTP score, CTP class and ethnicity (Table 4).

**Table 1:** Basic demographic data of study population

Age (in years)		46.89±11.9
Gender	Male	112 (56%)
	Female	88 (44%)
CTP class	B	102 (56%)
	C	98 (44%)
Ethnicity	Punjabi	31 (15.5%)
	Sindhi	55 (27.5%)
	Urdu	75 (37.5%)
	Baluchi	9 (4.5%)
	Pushto	19(9.5%)
	Others	11 (5.5%)
Mean BMI	With Gallstones	23.97 ± 4.41
	Without Gallstones	23.43 ± 4.76
Cause of CLD	Viral	155 (77.5%)
	Cryptogenic CLD	21 (10.5%)
	Autoimmune hepatitis	9 (4.5%)
	Wilson’s Disease	7 (3.5%)
	Hemochromatosis	1 (0.5%)
	Alcoholic CLD	5 (2.5%)
	Alcoholic +Viral CLD	1 (0.5%)
	Primary biliary cirrhosis	1 (0.5%)
Gall stones	Present Single	59 (29.5%) 19 (9.5%)
	Multiple	40 (20%)

**Table 2:** Stratification of factors with respect to the presence of gallstones

Factors		Gall stone present	Gallstone absent	p – value
Age	≤50 years	26 (32.9%)	53 (67.1)	0.43
	>50 years	33 (27.2%)	88 (72.8%)	
Gender	Male	33 (29.4%)	79 (70.6%)	1.0
	Female	26 (29.5%)	62 (70.5%)	
BMI (Kg/m2)	≤23.59	30 (27%)	81 (73%)	0.437
	>23.59	29 (32.5%)	60 (67.5%)	
Anti HCV	Positive	46 (34.5%)	87 (65.5%)	0.032
	Negative	13 (19.5%)	54 (80.5%)	
CTP score	≤9	22 (21.5%)	80 (78.5%)	0.013
	>9	37 (37.7%)	61 (62.3%)	
CTP class	B	22 (21.5%)	80 (78.5%)	0.013
	C	37 (37.7%)	61 (62.3%)	
Duration of Disease	>24 months	20 (25.3%)	59 (74.7%)	0.343
	≤24 months	39 (32.2%)	82 (61.8%)	
Gall stone number	Single	19 (9.5%)	141 (70.5%)	0.00
	Multiple	40 (20%)		
Ethnicity	Punjabi	7 (22.5%)	24 (77.5%)	0.260
	Sindhi	16 (29%)	39 (71%)	
	Urdu	25 (33.3%)	50 (66.7%)	
	Pathan	3 (15.7%)	16 (84.3%)	
	Balochi	2 (22.2%)	7 (77.8%)	
	Others	6 (45.5%)	5 (55.5%)	

**Table 3:** Stratification of factors with respect to the number of gall stones

Factor		No Gallstone	Single stone	Multiple stones	p – value
Age	≤50 years	53 (67%)	6 (7.5%)	20 (25.5%)	0.278
	>50 years	88 (72.7%)	13 (10.7%)	20 (16.6%)	
Gender	Male	79 (70.5%)	14 (12.5%)	19 (17%)	0.167
	Female	62 (70.4%)	5 (5.6%)	21 (24%)	
BMI (Kg/m2)	≤23.59	81 (72.9%)	13 (11.7%)	17 (15.4%)	0.120
	>23.59	60 (67.4%)	6 (6.7%)	23 (25.9%)	
Duration of CLD	>24 months	59 (74.6%)	6 (7.6%)	14 (17.8%)	0.559
	≤24 months	82 (67.7%)	13 (10.7%)	26 (21.6%)	
CTP score	≤9	80 (78.4%)	5 (5%)	17 (16.6%)	**0.022**
	>9	61 (62.2%)	14 (14.2%)	23 (23.6%)	
CTP class	B	80 (78.4%)	5 (5%)	17 (16.6%)	**0.022**
	C	61 (62.2%)	14 (14.2%)	23 (23.6%)	
Anti HCV	Present	87 (65.3%)	14 (10.7%)	32 (24%)	0.075
	Absent	54 (80.6%)	5 (7.4%)	8 (12%)	
Ethnicity	Punjabi	24 (77.5%)	1 (3.2%)	6 (19.3%)	0.249
	Sindhi	39 (71%)	3 (5.4%)	13 (23.6%)	
	Urdu	50 (66.7%)	12 (16%)	13 (17.3%)	
	Pathan	16 (84.3%)	1 (5.2%)	2 (10.5%)	
	Balochi	7 (77.8%)	0 (0%)	2 (22.2%)	
	Others	5 (45.5%)	2 (18.1%)	4 (36.4%)	

**Table 4:** Stratification of mean BMI in patients with or without gallstones with respect to effect modifiers

Factors		BMI ≤23.59 Kg/m^2^	BMI >23.59	p – value
Age	≤50 years	43 (54.4%)	36 (45.6%)	0.884
	>50 years	68 (56.2%)	53 (43.8%)	
Gender	Male	69 (61.6%)	43 (38.4%)	0.062
	Female	42 (47.7%)	46 (52.3%)	
Gall stone	Present	28 (47.4%)	31 (52.6%)	0.757
	Absent	72 (51%)	69 (49%)	
Duration of CLD	>24 months	45 (56.9%)	34 (43.1%)	0.772
	≤24 months	66 (54.5%)	55 (45.5%)	
CTP score	≤9	51 (50%)	51 (50%)	0.120
	>9	60 (61.2%)	38 (38.8%)	
CTP class	B	51 (50%)	51 (50%)	0.120
	C	60 (61.2%)	38 (38.8%)	
Anti HCV	Present	63 (47.3%)	70 (52.7%)	**0.001**
	Absent	48 (71.6%)	19 (28.4%)	
Ethnicity	Punjabi	16 (51.6%)	15 (49.4%)	0.668
	Sindhi	34 (61.8%)	21 (38.2%)	
	Urdu	42 (56%)	33 (44%)	
	Pathan	8 (42.1%)	11 (57.9%)	
	Balochi	4 (44.4%)	5 (55.6%)	
	Others	7 (63.6%)	4 (46.4%)	

## Discussion

Chronic liver disease is one of the major health problems globally, causing increased morbidity and mortality. In our country the most common cause of chronic liver disease is hepatitis C 28% followed by hepatitis B 22%, whereas alcoholic liver disease is more common etiologic agent worldwide [4]. This is also maintained in present study, viral etiology was found to be most common of decompensated liver disease, of which hepatitis C virus related was found to be predominant (66%). This disease results in various complications and is also considered an important risk factor for gall stone formation. Based on the findings of our study the frequency of gallstone formation in decompensated CLD was found to be 29.5% which is almost similar to the study based in Lahore by Naheed et al, who reported gall stones in 31% of patients [2]. This finding is also confirmed by various other studies done globally and the prevalence varied between 21 to 41% [18-20]. Acalovschi also mentioned that the incidence of gall stones is five times higher in ESLD [18,21]. In majority of our patients gallstones were not causing any symptoms and were the incidental finding on ultrasonography. The conventional risk factors of gall stone formation in general population and cirrhotic included female gender, obesity, advancing age, increased estrogen, SOD, portal hypertension and HCV infection [19,22-29]. This is contrary to our results in decompensated CLD patients where male to female incidence was found to be almost equal and this could be explained due to fact that the estrogen metabolism is altered in such patients which leads to increased gall stone formation in males as well. The frequency of gall stone formation in fertile women is also increased possibly due to the excessive secretion of cholesterol into bile due to the influence of estrogens, as after menopause this increased tendency was declined due to decreased estrogen levels. Fornari et al also suggested that higher estrogen and progesterone levels are also related to impaired gall bladder motility resulting in stasis of bile in pregnant females [22]. Many other factors could also be attributed to the increased tendency of GSD in cirrhotics. Chawla et al [23] reported autonomic neuropathy may occur in advanced cirrhosis leading to sphincter of Oddi dysfunction (SOD) ultimately causing stasis because of impaired gall bladder emptying. Sarin et al [24] also reported twice increased frequency of gallstones in CLD patients having portal hypertension. This can be explained by the increased venous hydrostsatic pressure leading to prolonged congestion and edema of gall bladder wall. Thick gall bladder wall again is contributory factor for reduced contractility of gall bladder. Li et al also concluded that decreased bile acid secretions also occur in CLD patients due to damaged bile ductules and diminished residual liver reserve causing further precipitation of cholesterol and thus forming stones [30]. In addition to hyperestrogenisim, chronic hemolysis occurring due to hypersplenisim can also be one of the reason as mentioned by Conte et al [31].

Advanced age was found to be significantly associated with increased GSD by Zhang et al [20] and this was also maintained by our study which revealed highest gallstones incidence in patients above 50 years of age, 33 out of 121 (27.2%) had gallstones but that was not found to be statistically significant. Furthermore, advanced cirrhosis was significantly associated with gall stone formation in our study. We reported gallstones in 62.7% of child class C patients as in conformity with other studies, including Fornari et al which revealed 49.3 % in child class C after 4 years of follow up [22] whereas this was in contrast to the study done by Naheed et al, reported 70% gallstones in CTP class B and 30% in class C [2]. The most important risk factor of GSD in decompensated cirrhosis was found to be hepatitis C virus infection which revealed stones in 36% of patients in our study. This is in accordance with other studies done by Bini and McGready [25], Stroffinlini [19] and Chen et al [26], all of which reported strong association of GSD with HCV infection. The probable reason provided by Hwang et al [28] was HCV may bind to apolipoprotein A1 thus causing hepatic steatosis and increasing cholesterol lithogenesis. Furthermore Lai et al [28] concluded that the presence of hepatitis C virus in biliary epithelium was contributory to gall bladder dysfunction. No association of gallstones could be elicited with BMI in our study and this finding was also consistent with the study done by Chen et al [26] and Li et al also reported similar findings [30]. This contrasts with the prevalence of gallstones in general population where obesity leading to increased BMI is considered important risk factor. This could be explained by the fact that increased weight in cirrhotic population could be attributed to accumulation of ascites and no general consensus exists regarding calculation of BMI in patients with mild, moderate or gross ascites. Similarly, Sheen et al also concluded BMI is much less important as a risk factor in decompensated cirrhosis [29]. The limitation of this study was exclusion of compensated cirrhosis that could have provided more evidence for comparison with decompensated cirrhosis. SOD and Portal hypertension are important risk factor of GSD as reported by Chawla et al and Sarin et al, was not assessed in our study [23, 24] Although ethnicity was considered in our study but it was a single center study based on study population of a city, so there is further need of large multicenter study to assess true frequency and prevalence of gallstones with respect to ethnicity in our country. Unfortunately very scarce data is available in this regard so the effect of dietary and environmental factors could not be assessed.

## Conclusion

Presence of cholelithiasis is not uncommon in patients with decompensated cirrhosis. Gallstone formation is associated with advanced stage of cirrhosis and hepatitis C Virus related chronic liver disease. Contrary to the established risk factors for gallstone formation, no association of gender, duration of disease, ethnicity or BMI was found in decompensated liver disease.

### What is known about this topic

- Gall stone disease is common in decompensated liver disease;
- Gall stone disease is common in females in general population;
- Obesity is a risk factor for gall stone formation in general population.

### What this study adds

- Gall stone formation is more common in HCV related ESLD;
- Increased BMI has no role in gall stone formation in ESLD;
- There is no statistical significance in gall stone formation in female in patient with ESLD.

## Competing interests

The authors declare no competing interests.
